# An 8-Year Survivor after Three-Time Hepatectomies for Metachronous Pancreatic Adenocarcinoma Liver Metastases

**DOI:** 10.70352/scrj.cr.25-0242

**Published:** 2026-01-06

**Authors:** Toshiro Masuda, Hirohisa Okabe, Takeshi Morinaga, Naoki Umezaki, Hidetoshi Nitta, Masahiko Hirota, Ryoichi Kurano, Toru Beppu, Hiroki Sugita

**Affiliations:** 1 Department of Surgery, Kumamoto Regional Medical Center, Kumamoto, Kumamoto, Japan; 2 Department of Surgery, Yamaga City Medical Center, Yamaga, Kumamoto, Japan; 3 Department of Pathology, Kumamoto Regional Medical Center, Kumamoto, Kumamoto, Japan

**Keywords:** pancreatic ductal adenocarcinoma, liver metastasis, liver resection

## Abstract

**INTRODUCTION:**

Recurrence of pancreatic ductal adenocarcinoma (PDAC) after primary resection remains a challenging condition, and operative indications for metachronous liver metastases are limited.

**CASE PRESENTATION:**

A 60-year-old woman with no elevation of carcinoembryonic antigen, carbohydrate antigen 19-9, and Duke pancreatic monoclonal antigen type 2 underwent a distal pancreatectomy for PDAC. Two years and 4 months after the primary surgery, metachronous liver metastases in liver segments 4 and 5 were diagnosed. After receiving 8 courses of bi-weekly gemcitabine plus nab-paclitaxel, a partial liver resection of segment 5 with radiofrequency ablation (RFA) of segment 4 was performed. A laparoscopic partial liver resection of segment 8 was performed for another liver metastasis 5 years and 2 months after the primary resection. An open partial liver resection of segment 8 was performed for the liver recurrence adjacent to the previous resection area 7 years and 2 months after the initial pancreatectomy. More than 8 years have passed since the initial pancreatic surgery, and she is currently alive without disease.

**CONCLUSION:**

Multidisciplinary treatment, including chemotherapy, resection, and RFA, may increase survival time in selected PDAC patients.

## Abbreviations

CA19-9
: carbohydrate antigen 19-9
CEA
: carcinoembryonic antigen
DUPAN-2
: Duke pancreatic monoclonal antigen type 2
PDAC
: pancreatic ductal adenocarcinoma
RFA
: radiofrequency ablation

## INTRODUCTION

Recurrence of pancreatic ductal adenocarcinoma (PDAC) after primary resection remains a challenging condition, and chemotherapy is mostly considered to be the first choice for treatment.^1–3)^ Patients with isolated metachronous lung oligometastases are reportedly good candidates for surgical resection.^4–6)^ However, the operative indication for metachronous liver metastases is limited.^2,7)^ According to a review article on surgically treated PDAC patients with metachronous liver metastases, the median interval from pancreas surgery to hepatectomy, overall survival after hepatectomy, and overall survival after initial pancreatectomy were 7.6 to 18.4, 11.4 to 31.0, and 24.5 to 40 months, respectively.^8)^

We report a patient who underwent hepatectomy 3 times for metachronous liver metastases and has survived more than 8 years after primary resection of PDAC.

## CASE PRESENTATION

**Fig. 1:**
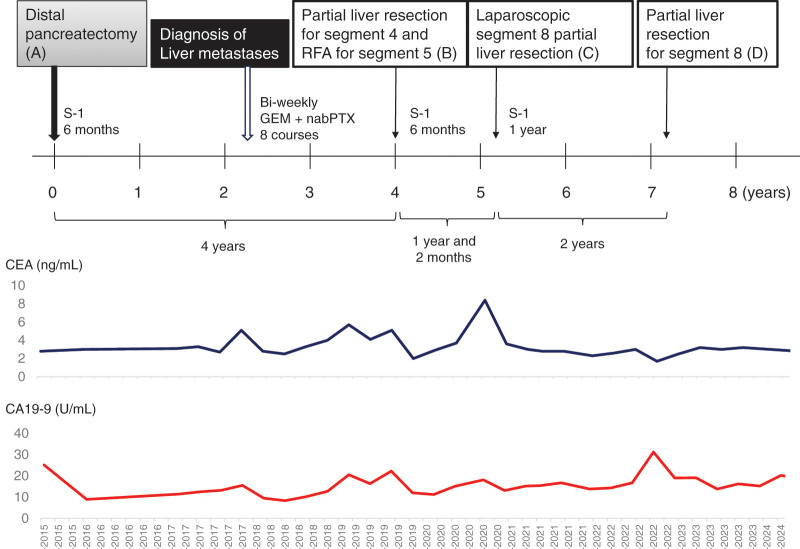
Clinical treatment course and the changes of tumor markers of the current patient. CA19-9, carbohydrate antigen 19-9; CEA, carcinoembryonic antigen 19-9; GEM, gemcitabine; nabPTX, nab-paclitaxel; RFA, radiofrequency ablation

**Fig. 2:**
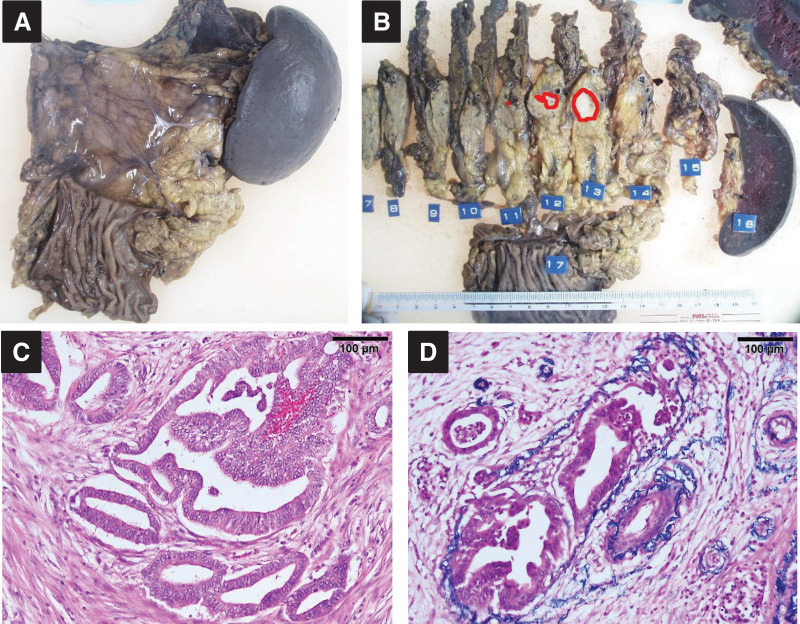
Resected specimen of distal pancreatectomy with transverse colon and left adrenal gland resection (**A**). Pancreatic adenocarcinoma was observed only in the circled area, and no invasion of the spleen, left adrenal gland, or transverse colon was seen (**B**). The diagnosis was well to moderately differentiated adenocarcinoma with v1 vascular invasion and no perineural invasion (**C**, **D**).

**Fig. 3:**
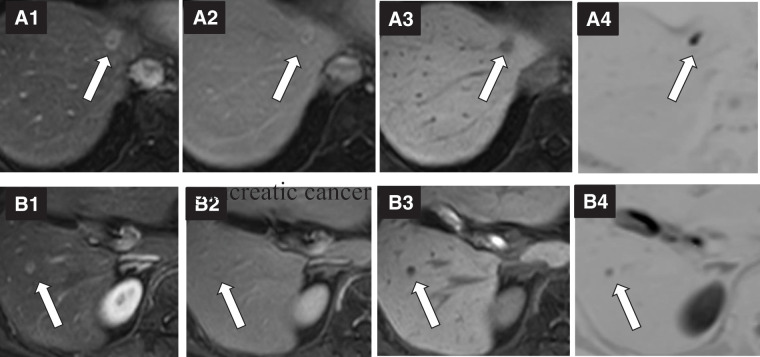
Gadolinium d-ethoxybenzyl-diethylenetriamine pentaacetic acid-enhanced MRI images of the first liver metastases. (**A**) A 10-mm tumor in liver segment 4 (arrow) and (**B**) a 7-mm tumor in liver segment 5 (arrow). (A1/B1) Early phase of T1-weighted dynamic MRI images; (A2/B2) delayed phase; (A3/B3) hepatobiliary phase; (A4/B4) diffusion-weighted MRI image.

**Fig. 4:**
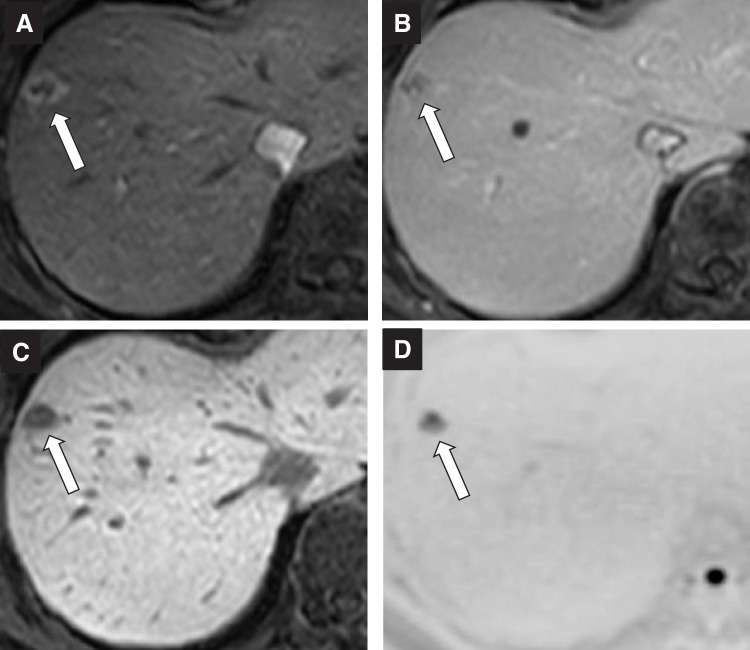
Gadolinium d-ethoxybenzyl-diethylenetriamine pentaacetic acid-enhanced MRI images of the second liver metastasis, measuring 10 mm in liver segment 8 (arrow). (**A**) The early phase of T1-weighted dynamic MRI image; (**B**) delayed phase; (**C**) hepatobiliary phase; and (**D**) diffusion-weighted MRI image.

**Fig. 5:**
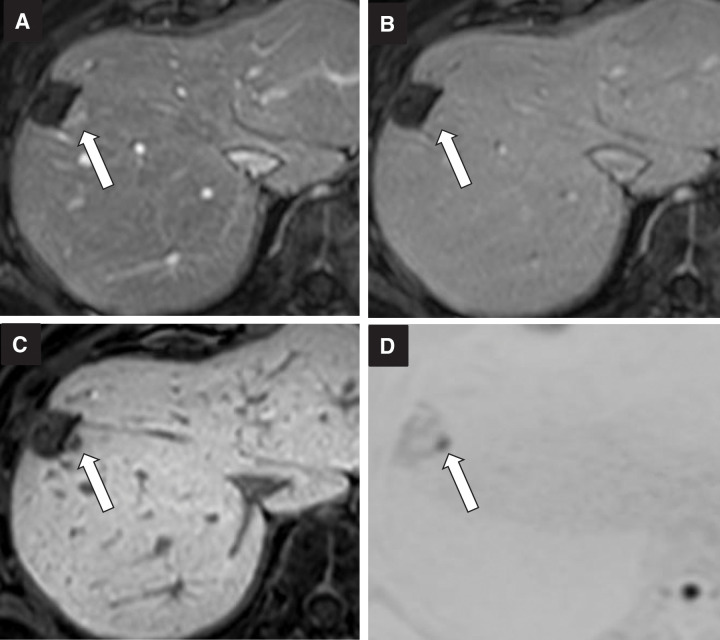
Gadolinium d-ethoxybenzyl-diethylenetriamine pentaacetic acid-enhanced MRI images of the third liver metastasis, measuring 7 mm adjacent to the lesion of the previous segment 8 liver resection (arrow). (**A**) The early phase of T1-weighted dynamic MRI image; (**B**) delayed phase; (**C**) hepatobiliary phase; and (**D**) diffusion-weighted MRI image.

**Fig. 6:**
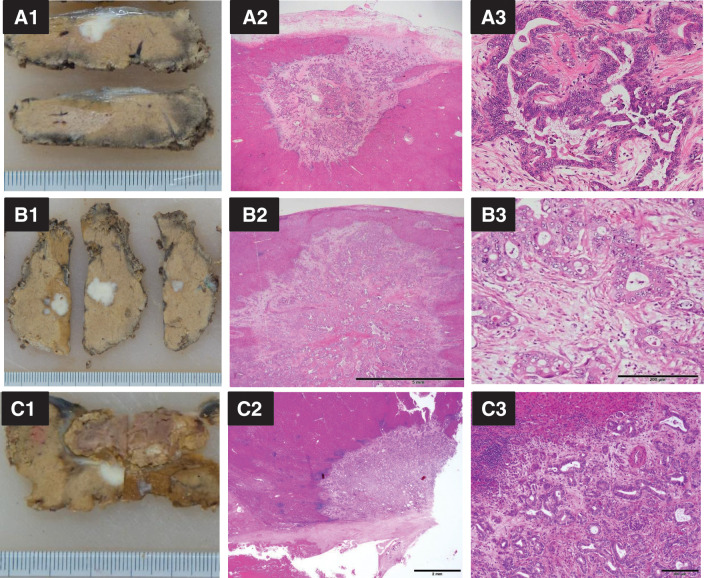
Macroscopic (1) and microscopic (2 and 3) findings. (**A**) Resected 1st liver tumor in liver segment 4; (**B**) 2nd liver metastasis in liver segment 8; (**C**) 3rd liver metastasis, adjacent to the lesion of the previous segment 8 liver resection.

A 60-year-old woman was referred to our hospital with a diagnosis of pancreatic tail cancer. Her carcinoembryonic antigen (CEA), carbohydrate antigen 19-9 (CA19-9), and Duke pancreatic monoclonal antigen type 2 (DUPAN-2) levels were within the normal range, 2.8 ng/mL, 25.1 U/mL, and <25 U/mL, respectively. She underwent a distal pancreatectomy with resection of the transverse colon and left adrenal gland (**Fig. 1A**). She experienced a mild postoperative pancreatic fistula, which subsequently improved, allowing discharge on POD 39. The diagnosis was well to moderately differentiated adenocarcinoma with v1 vascular invasion, without perineural invasion, or invasion of the spleen, left adrenal gland, or transverse colon (**Fig. 2**). The cancer stage was pT1N0M0 pStage I. As adjuvant chemotherapy, S-1 was administered for 6 months. Postoperative surveillance was conducted according to our institutional follow-up protocol, including blood tests with tumor markers (CEA, CA19-9) and imaging every 3 months using contrast-enhanced CT covering the chest/abdomen/pelvis, gadolinium d-ethoxybenzyl-diethylenetriamine pentaacetic acid-enhanced MRI, or abdominal ultrasound sonography. Two years and 4 months after the primary surgery, 2 metachronous liver metastases in segments 4 and 5 were diagnosed. CEA and CA19-9 levels were 2.3 ng/mL and 8.4 U/mL, respectively. Eight courses of bi-weekly gemcitabine plus nab-paclitaxel were administered. Since the segment 4 lesion was located at the liver surface and the segment 5 lesion was small and located deep in the liver >5 mm away from the Glissonian pedicle, a partial liver resection was performed for the 10-mm tumor in segment 4 (**Fig. 3A**), combined with radiofrequency ablation (RFA) for the 7-mm tumor in segment 5 (**Fig. 3B**) 4 years after the initial pancreatectomy (**Fig. 1B**). Additional adjuvant chemotherapy with S-1 for 6 months was applied, and another metachronous liver metastasis, 10 mm in diameter, was diagnosed in segment 8 (**Fig. 4**). CEA, CA19-9, and DUPAN-2 levels were 8.4 ng/mL, 18.1 U/mL, and 59 U/mL, respectively. An upfront laparoscopic partial liver resection of segment 8 was performed 1 year and 2 months after the 1st liver resection (5 years and 2 months after the initial pancreatectomy) (**Fig. 1C**). Another adjuvant chemotherapy with S-1 was performed for 1 year. Two years after the 2nd liver resection, 7 years and 2 months after the initial pancreatectomy, a 7-mm metachronous liver metastasis adjacent to the lesion of the previous segment 8 liver resection site was diagnosed (**Fig. 5**). CEA and CA19-9 levels were 3.0 ng/mL and 15.4 U/mL, respectively. An open partial liver resection of segment 8 was performed (**Fig. 1D**). After the 3rd liver surgery, another course of adjuvant chemotherapy using S-1 was started but was discontinued because of the adverse events such as fatigue and appetite loss. Macroscopic and microscopic findings of the resected specimens from the 3 liver resections are shown in **Fig. 6A**–**6C**, respectively. More than 8 years have passed after the initial pancreatic surgery, and she is currently alive without disease. The patient’s clinical course and changes in tumor markers are shown in **Fig. 1**. Histopathological findings of the primary pancreatic cancer and liver metastases are shown in **Supplementary Table 1**.

## DISCUSSION

FOLFIRINOX,^9)^ nab-paclitaxel plus gemcitabine,^10)^ and newer modalities of treatment, including targeted therapies,^3)^ have improved outcomes in metastatic PDAC. With the development of chemotherapy, the number of patients who undergo simultaneous resection for synchronous PDAC liver metastases^11–14)^ or liver resection for metachronous oligometastatic liver recurrence after primary PDAC resection^11)^ has been increasing.

There have been some reports suggesting that liver resection for metachronous liver metastases can be beneficial in selected pancreatic cancer patients. Dünschede et al. reported that in patients with metachronous liver metastases, the median survival was increased after liver resection compared to patients who were treated with gemcitabine (31 vs. 11 months).^15)^ Schwarz et al. reported a retrospective comparison between patients with metachronous PDAC liver metastases with liver resection versus chemotherapy only.^16)^ Median overall survival was 36.8 months in the liver resection group versus 9.2 months in the chemotherapy-only group (P = 0.0007). Shibata et al. suggested that liver resection may be a treatment option for patients with solitary liver recurrence after PDAC resection.^17)^ Saito et al. investigated the prognostic factors in patients with oligometastatic liver recurrence after PDAC surgery and identified short recurrence-free survival (<6 months), short stable disease interval (≤3 months), and 4 or more recurrent tumors as independent poor prognostic factors.^18)^ Besides, the outcome of patients with no elevation of the 3 preoperative tumor markers, including CEA, CA19-9, and DUPAN-2, who underwent primary resection is reportedly better than that of those with any marker elevated.^19,20)^ In the current case, preoperative CEA, CA19-9, and DUPAN-2 were within normal ranges. Throughout the patient’s clinical course, CEA and CA19-9 mostly remained normal, and only CEA increased to 8.4 ng/mL at the time of diagnosis of the second metachronous liver metastasis in segment 8. This level is not high enough to suggest an association with cancer recurrence. Besides, the metachronous liver metastases of PDAC were oligometastatic, and the recurrence-free interval was very long, 2 years and 4 months. These factors might affect the good outcome of this patient. At the time of first liver metastases, 8 courses of gemcitabine plus nab-paclitaxel were administered, and we ascertained that there was no increase in the number or size of her liver metastases, so we chose a liver resection. Previously reported cases of long-term survival after hepatectomy for pancreatic cancer liver metastases are reviewed in **Table 1**.^21–27)^ Notably, the current patient achieved long-term survival after undergoing hepatectomy 3 times for metachronous liver metastases from pancreatic cancer.

**Table 1:** Reported cases of long-term survival after surgical resection of pancreatic cancer liver metastases

Authors	Year	Age	Sex	Primary operation	Liver metastasis timing	Number of sessions of liver resection	Survival from the primary resection (year)
Iida et al.	2014	63	Female	PD	Metachronous	1	>6
Fujisaki et al.	2017	51	Male	PD	Metachronous	1	>5
Shimura et al.	2017	73	Male	DP-CAR	Synchronous	1	>4.5
Tsutsumi et al.	2020	51	Male	PD	Metachronous	1	>11
Sato et al.	2020	42	Female	DP	Metachronous	1	>8
Obed et al.	2023	56	Male	DP	Synchronous	2	3.5
Tohyama et al.	2024	70	Male	DP	Metachronous	1	>5.5
Present case	2025	60	Female	DP	Metachronous	3	>8

DP-CAR, distal pancreatectomy with celiac axis resection; PD, pancreaticoduodenectomy

Following the recommendation of the Japanese clinical practice guidelines for pancreatic cancer, S-1 was used as adjuvant chemotherapy not only after the primary resection but also after the resections of liver metastases.^2)^ In the JASPAC-01 study, recurrence-free survival for patients receiving adjuvant S-1 was especially good, at 22.9 months.^28)^ Adjuvant S-1 might affect the long disease-free intervals after the surgeries for the primary tumor and liver metastases in the current patient. Other adjuvant therapy regimens, such as gemcitabine plus capecitabine^29)^ or FOLFIRINOX,^30)^ may also be beneficial after surgeries for both primary pancreatic cancer and liver metastases.

RFA is increasingly used to treat oligometastatic liver disease not only from colorectal cancer but also from PDAC.^31)^ Lee et al. reported that in patients with metachronous PDAC liver metastases, those with small diameter tumors, early TNM stage before primary surgery, late hepatic recurrence, and liver-only metastasis benefit most from RFA treatment.^32)^ In our case, RFA was applied to a 7-mm metachronous liver metastasis. More than 4 years have passed after applying RFA, and there has been no local recurrence in this region.

Recently, a survival benefit of stereotactic body radiation therapy (SBRT) plus standard chemotherapy for oligometastatic pancreatic cancer has been reported.^33)^ However, no improvement in overall or recurrence-free survival was observed in SBRT for pancreatic cancer liver metastases.^33)^

## CONCLUSION

We experienced an extremely rare, long-surviving PDAC patient who underwent a primary resection and 3-time hepatectomies with an RFA for metachronous liver metastases. Multidisciplinary treatment, including chemotherapy, resection, and RFA, may increase survival time in selected PDAC patients.

## SUPPLEMENTARY MATERIALS

Supplementary Table 1 Histopathological findings of primary pancreatic cancer and liver metastases

## DECLARATIONS

### Availability of data and materials

Data will be made available on reasonable request.

## References

[1] Conroy T, Pfeiffer P, Vilgrain V, et al. Pancreatic cancer: ESMO Clinical Practice Guideline for diagnosis, treatment and follow-up. Ann Oncol 2023; 34: 987–1002. 37678671 10.1016/j.annonc.2023.08.009

[2] Okusaka T, Nakamura M, Yoshida M, et al. Clinical Practice Guidelines for Pancreatic Cancer 2022 from the Japan Pancreas Society: a synopsis. Int J Clin Oncol 2023; 28: 493–511. 36920680 10.1007/s10147-023-02317-x PMC10066137

[3] Tempero MA, Malafa MP, Al-Hawary M, et al. Pancreatic Adenocarcinoma, Version 2.2021, NCCN Clinical Practice Guidelines in Oncology. J Natl Compr Canc Netw 2021; 19: 439–57. 33845462 10.6004/jnccn.2021.0017

[4] Lovecek M, Skalicky P, Chudacek J, et al. Different clinical presentations of metachronous pulmonary metastases after resection of pancreatic ductal adenocarcinoma: Retrospective study and review of the literature. World J Gastroenterol 2017; 23: 6420–8. 29085191 10.3748/wjg.v23.i35.6420 PMC5643267

[5] Kruger S, Haas M, Burger PJ, et al. Isolated pulmonary metastases define a favorable subgroup in metastatic pancreatic cancer. Pancreatology 2016; 16: 593–8. 27067420 10.1016/j.pan.2016.03.016

[6] Groot VP, Blair AB, Gemenetzis G, et al. Isolated pulmonary recurrence after resection of pancreatic cancer: the effect of patient factors and treatment modalities on survival. HPB (Oxford) 2019; 21: 998–1008. 30777697 10.1016/j.hpb.2018.12.002

[7] Yamamoto M, Yoshida M, Furuse J, et al. Clinical practice guidelines for the management of liver metastases from extrahepatic primary cancers 2021. J Hepatobiliary Pancreat Sci 2021; 28: 1–25. 33200538 10.1002/jhbp.868

[8] Sakaguchi T, Valente R, Tanaka K, et al. Surgical treatment of metastatic pancreatic ductal adenocarcinoma: A review of current literature. Pancreatology 2019; 19: 672–80. 31285145 10.1016/j.pan.2019.05.466

[9] Conroy T, Desseigne F, Ychou M, et al. FOLFIRINOX versus gemcitabine for metastatic pancreatic cancer. N Engl J Med 2011; 364: 1817–25. 21561347 10.1056/NEJMoa1011923

[10] Von Hoff DD, Ervin T, Arena FP, et al. Increased survival in pancreatic cancer with nab-paclitaxel plus gemcitabine. N Engl J Med 2013; 369: 1691–703. 24131140 10.1056/NEJMoa1304369 PMC4631139

[11] Halle-Smith JM, Powell-Brett S, Roberts K, et al. Resection of isolated liver oligometastatic disease in pancreatic ductal adenocarcinoma: Is there a survival benefit? A systematic review. World J Gastrointest Surg 2023; 15: 1512–21. 37555114 10.4240/wjgs.v15.i7.1512 PMC10405113

[12] Voss N, Izbicki JR, Nentwich MF. Oligometastases in pancreatic cancer (Synchronous resections of hepatic oligometastatic pancreatic cancer: Disputing a principle in a time of safe pancreatic operations in a retrospective multicenter analysis). Ann Gastroenterol Surg 2019; 3: 373–7. 31346576 10.1002/ags3.12255 PMC6635688

[13] Hackert T, Niesen W, Hinz U, et al. Radical surgery of oligometastatic pancreatic cancer. Eur J Surg Oncol 2017; 43: 358–63. 27856064 10.1016/j.ejso.2016.10.023

[14] Koti S, Demyan L, Deutsch G, et al. Surgery for oligometastatic pancreatic cancer: defining biologic resectability. Ann Surg Oncol 2024; 31: 4031–41. 38502293 10.1245/s10434-024-15129-8 PMC11076395

[15] Dünschede F, Will L, von Langsdorf C, et al. Treatment of metachronous and simultaneous liver metastases of pancreatic cancer. Eur Surg Res 2010; 44: 209–13. 20571276 10.1159/000313532

[16] Schwarz C, Fitschek F, Primavesi F, et al. Metachronous hepatic resection for liver only pancreatic metastases. Surg Oncol 2020; 35: 169–73. 32889249 10.1016/j.suronc.2020.08.005

[17] Shibata Y, Uemura K, Sumiyoshi T, et al. Surgical resection for liver recurrence after curative resection of pancreatic ductal adenocarcinoma. Langenbecks Arch Surg 2023; 408: 280. 37458812 10.1007/s00423-023-03009-w

[18] Saito R, Ban D, Mizui T, et al. Oligo-like liver metastasis: a novel prognostic indicator to improve survival in pancreatic cancer. Ann Gastroenterol Surg 2023; 8: 481–9. 38707220 10.1002/ags3.12753 PMC11066487

[19] Miyata T, Hayashi H, Yamashita YI, et al. Prognostic value of the preoperative tumor marker index in resected pancreatic ductal adenocarcinoma: a retrospective single-institution study. Ann Surg Oncol 2021; 28: 1572–80. 32804325 10.1245/s10434-020-09022-3

[20] Takagi T, Nagai M, Nishiwada S, et al. Importance of triple tumor markers as biomarkers in patients with pancreatic ductal adenocarcinoma. Ann Gastroenterol Surg 2022; 7: 326–35. 36998299 10.1002/ags3.12629 PMC10043775

[21] Iida T, Nakabayashi Y, Okui N, et al. Successful management of metachronous liver metastasis after pancreaticoduodectomy for pancreatic ductal carcinoma using hepatectomy and chemotherapy: a case report. Anticancer Res 2014; 34: 2417–20. 24778053

[22] Fujisaki S, Takashina M, Tomita R, et al. Long-term survival following hepatectomy, radiation, and chemotherapy for recurrent pancreatic carcinoma: a case report. World J Surg Oncol 2017; 15: 157. 28835248 10.1186/s12957-017-1232-2 PMC5569547

[23] Shimura M, Mizuma M, Hayashi H, et al. A long-term survival case treated with conversion surgery following chemotherapy after diagnostic metastasectomy for pancreatic cancer with synchronous liver metastasis. Surg Case Rep 2017; 3: 132. 29285651 10.1186/s40792-017-0409-9 PMC5746493

[24] Tsutsumi C, Abe T, Shinkawa T, et al. Long-term survival after hepatectomy for metachronous liver metastasis of pancreatic ductal adenocarcinoma: a case report. Surg Case Rep 2020; 6: 157. 32621095 10.1186/s40792-020-00924-8 PMC7334331

[25] Sato H, Sasajima J, Okada T, et al. Resection for pancreatic cancer metastases contributes to survival: a case report with sequential tumor genotype profiling during the long-term postoperative course. Medicine (Baltimore) 2020; 99: e20564. 32569179 10.1097/MD.0000000000020564 PMC7310851

[26] Obed A, Siyam M, Jarrad AA, et al. Unexpected long-term survival of Stage IV pancreatic cancer patient with synchronic liver metastases after multimodal therapy including upfront surgery. J Surg Case Rep 2023; 2023: rjac638. 36636652 10.1093/jscr/rjac638 PMC9831652

[27] Tohyama T, Tanno Y, Murakami T, et al. A case of metachronous oligo-hepatic and peritoneal metastases of pancreatic cancer with a favorable outcome after conversion surgery combined with perioperative sequential chemotherapy. Clin J Gastroenterol 2024; 17: 371–81. 38291249 10.1007/s12328-023-01917-4

[28] Uesaka K, Boku N, Fukutomi A, et al. Adjuvant chemotherapy of S-1 versus gemcitabine for resected pancreatic cancer: a phase 3, open-label, randomised, non-inferiority trial (JASPAC 01). Lancet 2016; 388: 248–57. 27265347 10.1016/S0140-6736(16)30583-9

[29] Neoptolemos JP, Palmer DH, Ghaneh P, et al. Comparison of adjuvant gemcitabine and capecitabine with gemcitabine monotherapy in patients with resected pancreatic cancer (ESPAC-4): a multicentre, open-label, randomised, phase 3 trial. Lancet 2017; 389: 1011–24. 28129987 10.1016/S0140-6736(16)32409-6

[30] Conroy T, Castan F, Lopez A, et al. Five-year outcomes of FOLFIRINOX vs gemcitabine as adjuvant therapy for pancreatic cancer: a randomized clinical trial. JAMA Oncol 2022; 8: 1571–8. 36048453 10.1001/jamaoncol.2022.3829 PMC9437831

[31] Ghidini M, Petrillo A, Salati M, et al. Surgery or locoregional approaches for hepatic oligometastatic pancreatic cancer: myth, hope, or reality? Cancers (Basel) 2019; 11: 1095. 31374916 10.3390/cancers11081095 PMC6721290

[32] Lee SJ, Kim JH, Kim SY, et al. Percutaneous radiofrequency ablation for metachronous hepatic metastases after curative resection of pancreatic adenocarcinoma. Korean J Radiol 2020; 21: 316–24. 32090524 10.3348/kjr.2019.0647 PMC7039725

[33] Elhariri A, Patel J, Mahadevia H, et al. Stereotactic body radiation therapy in oligometastatic pancreatic cancer: overall survival improvement and SMAD4 as a predictor of progression-free survival. J Gastrointest Oncol 2025; 16: 1658–66. 40950337 10.21037/jgo-2025-100 PMC12432913

