# Supplementary material for: An 8-Year Survivor after Three-Time Hepatectomies for Metachronous Pancreatic Adenocarcinoma Liver Metastases
Source: Surg Case Rep. 2026 Jan 6;12(1):25-0242. doi: 10.70352/scrj.cr.25-0242 (PMC12779356; doi:10.70352/scrj.cr.25-0242)
Supplement: Supplementary Table 1 — Histopathological findings of primary pancreatic cancer and liver metastases [file scr-12-01-25-0242-s001.pdf]

Supplementary Table 1 Histopathological findings of primary pancreatic cancer and liver metastases

	Tumor differentiation	Surgical margin	Vascular or perineural invasion
Primary pancreatic cancer	well to moderate	R0	v1 vascular invasion and no perineural invasion
The first liver metastasis (S4)	well	R0	no vascular or perineural invasion
The second liver metastatis (S8)	well to moderate	R0	no vascular or perineural invasion
The third liver metastasis (S8)	well to moderate	R0	no vascular or perineural invasion
